# Event and Comment

**Published:** 1918-06

**Authors:** 


### DENTAL REGISTER

Vol. LXXII JUNE, 1918 No. 6

### EVENT AND COMMENT

**Meritorious Recognition** The French government has bestowed upon Dr. George B. Hayes and his confrere, Dr. Wm. Davenport, the honorable decorations of "Chevalier of the Legion of Honor," for the invaluable work they have done in serving the wounded French soldiers through the American Red Cross hospital at Nuilly, France. All American dentists know of the wonderful work done in facial restorations by these dentists in this hospital, but few know of the tremendous sacrifices of time and physical strength that this has involved. We are not only proud that the French have in such a proper manner made acknowledgment of this work, but we should be glad to honor the men who have made known the fine American spirit in this great crisis of moral development. There should be some proper recognition of this service by these faithful professional representatives of American dentistry in our national organizations.

---

**Army Medical Museum and Library** This museum and library is undergoing some radical changes in the way of recataloging and classification so as to make it available to the different classes of people who may have use for its material. Surgeon General Gorgas has recently detailed Major R. W. Shufeldt to the task of this reconstruction. The museum has a considerable collection of dental interest and it is desired that this may be made as complete as possible. Anyone who can contribute to this end will help to make this museum and library what it should be, a place for preserving and consulting the historical archives of our profession. If you are interested and can make any contributions to this cause write to Major R. W. Shufeldt, Army Medical Museum and Library, Washington, D. C.

---

**Prosthesis for Oral Mutilations** In studying the illustrations of cases treated by dentists in the great war we have noticed the very complex appliances that have been used in many cases, and the suggestion has come to us, that a simplification of the technical forms would be desirable if the requirements could be met. Many of the dentists who are taking up this war work are inexpert as technical prosthetists because of lack of experience, and besides they have had little occasion to study the phenomena involved, so that they could have any very well-defined ideas of the principles involved in designing and constructing suitable appliances for these entirely new conditions. The fundamental objectives to be kept in view are the restoration of the functions of mastication and speech, and so far as may be practicable of normal facial contour.

Probably the most difficult technical problem will be to secure stability of the appliances that shall make functions practicable. In some cases the tissues are badly mutilated, and in many cases scar tissue from healing wounds has not only changed the usual form of the oral structures, but has produced conditions which make it difficult to adapt any of the usual forms of prosthetic dentures. Many or all of the teeth and large sections of the jaw bones are often missing from gun shot wounds or because of the surgeon's operations, and the remaining tissues are of such character and condition that little form or surface remains to which definite anchorage may be attached. What is still more troublesome is the fact that the muscles of mastication are destroyed by the wounds, or by paralysis, so that the harmony of masticating function is badly deranged. From this brief survey it becomes very evident that it requires some mechanical ingenuity, or wise consideration of the anatomical and physiological disarrangements to even begin to design an appropriate appliance. We are wondering where there is to be found a man or an institution that will take up a careful study of this problem at once so that some adequate instruction may be made available to every dental surgeon before he goes across, along the line of facial restorations, that will be acceptable and within the range of the technical skill of the average dentist. It is a problem that is worthy of our best effort and it would seem that it should be capable of a satisfactory solution. The physics and mechanics as well as the functional requirements should be worked out together, as no real success can be predicted without a harmonious control of all the forces involved. It may be that it will be time enough to meet this problem when our wounded soldiers return, but in the meantime it would seem that it would be wise to begin some serious study of this problem that we may save our men from the inconvenience of lack of function, or the embarrassment of personal disfigurement.

